# Identification of a Five-Autophagy-Related-lncRNA Signature as a Novel Prognostic Biomarker for Hepatocellular Carcinoma

**DOI:** 10.3389/fmolb.2020.611626

**Published:** 2021-01-11

**Authors:** Xiaoyu Deng, Qinghua Bi, Shihan Chen, Xianhua Chen, Shuhui Li, Zhaoyang Zhong, Wei Guo, Xiaohui Li, Youcai Deng, Yao Yang

**Affiliations:** ^1^Institute of Materia Medica, College of Pharmacy, Army Medical University (Third Military Medical University), Chongqing, China; ^2^Department of Hepatobiliary Surgery, The First Affiliated Hospital of Army Medical University (Third Military Medical University), Chongqing, China; ^3^Diagosis and Treatment Center for Servicemen, The First Affiliated Hospital of Army Medical University (Third Military Medical University), Chongqing, China; ^4^Department of Clinical Biochemistry, Faculty of Pharmacy and Laboratory Medicine, Army Medical University (Third Military Medical University), Chongqing, China; ^5^Cancer Center, Daping Hospital and Research Institute of Surgery, Army Medical University (Third Military Medical University), Chongqing, China; ^6^Department of Pharmacy, Southwest Hospital, Third Military Medical University, Chongqing, China

**Keywords:** hepatocellular carcinoma, prognostic signature, long non-coding RNA, autophagy, stratification analysis, autophagy-related long non-coding RNA

## Abstract

Although great progresses have been made in the diagnosis and treatment of hepatocellular carcinoma (HCC), its prognostic marker remains controversial. In this current study, weighted correlation network analysis and Cox regression analysis showed significant prognostic value of five autophagy-related long non-coding RNAs (AR-lncRNAs) (including TMCC1-AS1, PLBD1-AS1, MKLN1-AS, LINC01063, and CYTOR) for HCC patients from data in The Cancer Genome Atlas. By using them, we constructed a five-AR-lncRNA prognostic signature, which accurately distinguished the high- and low-risk groups of HCC patients. All of the five AR lncRNAs were highly expressed in the high-risk group of HCC patients. This five-AR-lncRNA prognostic signature showed good area under the curve (AUC) value (AUC = 0.751) for the overall survival (OS) prediction in either all HCC patients or HCC patients stratified according to several clinical traits. A prognostic nomogram with this five-AR-lncRNA signature predicted the 3- and 5-year OS outcomes of HCC patients intuitively and accurately (concordance index = 0.745). By parallel comparison, this five-AR-lncRNA signature has better prognosis accuracy than the other three recently published signatures. Furthermore, we discovered the prediction ability of the signature on therapeutic outcomes of HCC patients, including chemotherapy and immunotherapeutic responses. Gene set enrichment analysis and gene mutation analysis revealed that dysregulated cell cycle pathway, purine metabolism, and TP53 mutation may play an important role in determining the OS outcomes of HCC patients in the high-risk group. Collectively, our study suggests a new five-AR-lncRNA prognostic signature for HCC patients.

## Introduction

Hepatocellular carcinoma (HCC) is a kind of malignant neoplasm that is the sixth most commonly diagnosed cancer and the fourth leading cause of cancer-related death worldwide (Singal et al., 2020). Although great developments have been made in the treatment of HCC (Feng et al., 2020), its prognosis remains poor. Tumor extent, severity of liver dysfunction, and general health status of patients were confirmed as key predictors for HCC prognosis; however, the heterogeneity of HCC patients affected the accuracy and applicable scope of the current existing prediction methods (Liu et al., 2016). Therefore, new biomarkers with improved prediction efficiency are urgently necessary for the prognosis of HCC.

Autophagy describes a conserved cellular process that degrades the damaged and mutated cytoplasmic materials by lysosomes so as to maintain the cellular homeostasis under physiological or pathological conditions (Jiang and Mizushima, 2014). Previous studies have revealed that autophagy played various roles in different stages of HCC development (Gerada and Ryan, 2020). Several autophagy-related genes (ARGs), such as LC3 and ULK1, have become emerging biomarkers to predict the prognosis of HCC (Wu et al., 2018; Meng et al., 2020). However, messenger RNA could display unsatisfied prediction because of its low tissue specificity (Deveson et al., 2017) and instability *in vivo* and *in vitro* (Tombacz et al., 2021). Hence, it is still critical to develop novel autophagy-related biomarkers for the prognosis of HCC.

Long non-coding RNA (lncRNA) is a kind of powerful biological functional non-coding RNA, which is longer than 200 nucleotides (Derrien et al., 2012). Sixty-eight percent of human cell transcripts are classified as lncRNAs (Han and Chang, 2015), which play irreplaceable roles in many biological processes (Chen et al., 2017). A large number of lncRNAs were previously found dysregulated in HCC (Cui et al., 2017). Recently, Sun et al. have comprehensively summarized the relationship between autophagy-related lncRNAs (AR-lncRNAs) and HCC. They reported that several AR-lncRNAs participated in the progression of HCC by regulating the expression of autophagy-related proteins, such as ATG3, ATG7, USP22, SIRT1, and PTEN (Sun, 2018). Given that some lncRNAs have been proven much more specific than other biomarker in cancer (Soares et al., 2019), it remains unknown whether a prognostic model composed of multiple AR-lncRNAs could act more efficiently than the current known prognostic signatures for HCC.

**Figure 1:**
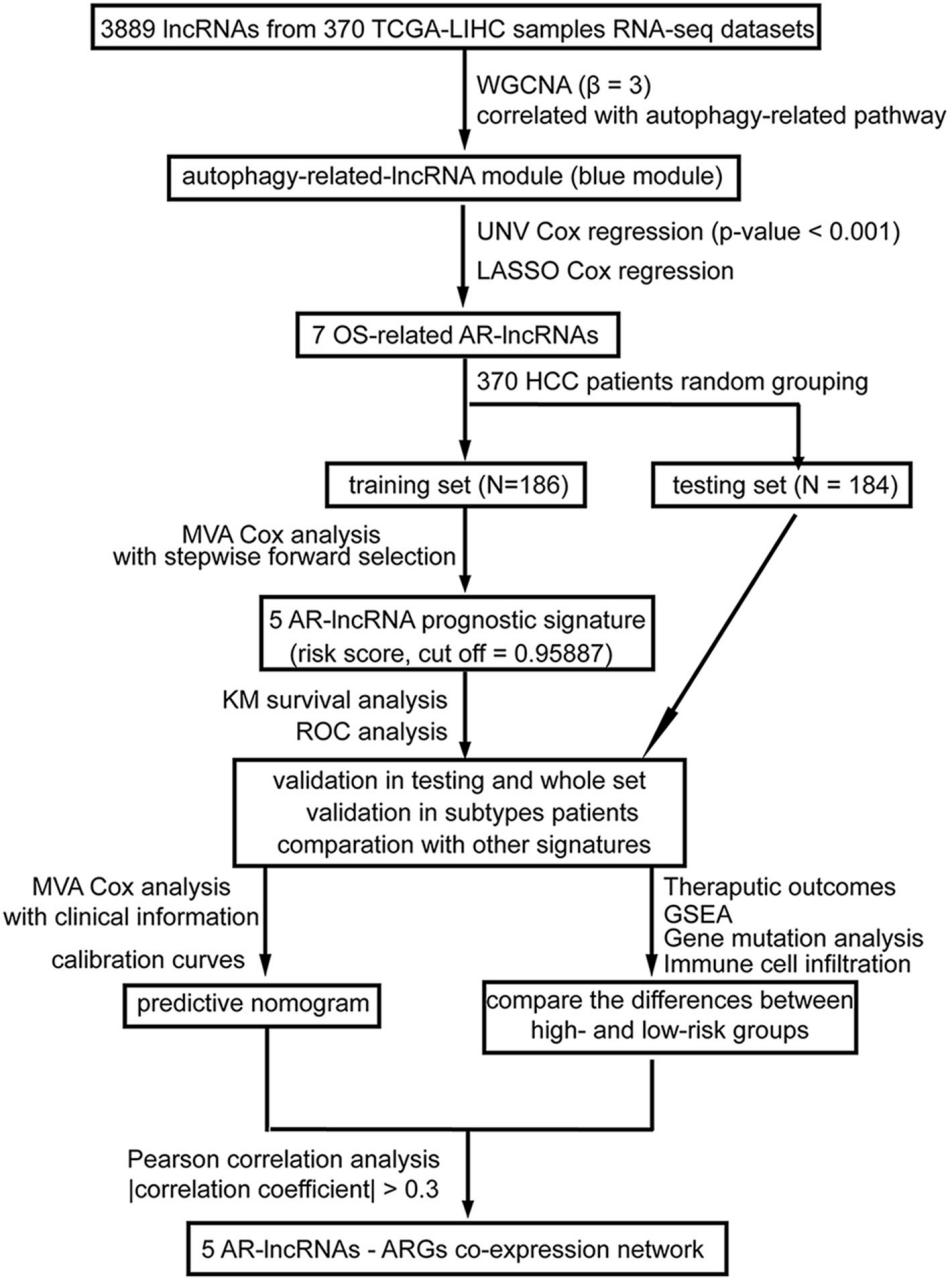
A workflow of the processing. The flowchart indicates the establishment, validation, and potential mechanism exploration of the autophagy-related lncRNA (AR-lncRNA) signature. WGCNA, weighted correlation network analysis; UNV Cox regression, univariate Cox regression; LASSO Cox analysis, least absolute shrinkage and selection operator Cox regression analysis; OS, overall survival; AR-lncRNA, autophagy-related lncRNA; MVA Cox regression, multivariate Cox regression; KM survival analysis, Kaplan–Meier survival analysis; ROC analysis, receiver operating characteristic curve analysis; GSEA, gene set enrichment analysis.

As shown in Figure 1, in the present study, after applying weighted correlation network analysis (WGCNA) and several kinds of Cox regression analysis on the database of HCC patients in The Cancer Genome Atlas (TCGA), five AR lncRNAs (TMCC1-AS1, PLBD1-AS1, MKLN1-AS, LINC01063, and CYTOR) were identified to construct a prognostic signature for the overall survival (OS) outcomes of HCC patients. The sensitivity and specificity of the five-AR-lncRNA signature surpassed three recently published prognostic signatures for HCC (Wang et al., 2017; Huo et al., 2020; Yang et al., 2020). Furthermore, significant differences were found in the therapeutic outcomes, including immunotherapy and chemotherapy responses, between the high- and low-risk groups. The distinction in prognosis between the high- and low-risk groups may partially due to the differences in the expression levels of ARGs correlated with these five AR lncRNAs.

## Methods

### Data Sources

The raw count RNA-seq data were downloaded from the TCGA–liver hepatocellular carcinoma (LIHC) dataset in the UCSC Xena (https://xenabrowser.net/datapages/) (Tomczak et al., 2015). The datasets contained a total of 424 samples (including 374 tumor samples and 50 non-tumor liver tissues), along with the corresponding clinical data [including 370 patients with OS information corresponding follow-up data and 319 patients with disease-free survival (DFS) corresponding follow-up data]. Patients with complete clinical prognostic data were included in the subsequent prognostic analysis.

Average raw read count >1 was applied to determine candidate genes that were reasonably expressed. Then, the raw count data were normalized by transcripts-per-million method and underwent a log2 transformation. LncRNAs were reannotated by gene symbol based on the gene annotation file “gencode.v35.long_noncoding_RNAs,” which was downloaded from the GENCODE website (https://www.gencodegenes.org/human/) (Liu et al., 2019b). ARGs were obtained from the HADb (Human Autophagy Database, http://www.autophagy.lu/). A total of 222 ARGs with expression value were obtained.

### WGCNA

WGCNA package (Langfelder and Horvath, 2008) (version 1.60) in R was used to find highly correlated lncRNAs to combine lncRNA modules and to search the relationship between each module and each clinical trait of the 370 HCC tumor sample with OS information in TCGA database (Mo et al., 2019). Here, the power of β = 3 (scale free *R*^2^ = 0.85) was selected as the soft threshold to ensure a scale-free network (Supplementary Figure 1). The dynamic tree cutting method was used to cluster the lncRNAs in layers, using 50 as a minimum size cutoff, and the cut height = 0.3 was applied to merge highly similar modules. Different lncRNA modules were labeled with different colors, and the gray module contained lncRNAs that cannot be merged. Pearson correlation analysis was applied to evaluate the correlation between lncRNAs in each module and each clinical feature. Autophagy pathway values of each HCC case were estimated by the gene set variation analysis (Liu et al., 2020), and the most relevant module related to autophagy was selected for further analysis.

### Establishment and Verification of AR-lncRNA Signature

First, the univariate Cox regression analysis was used to evaluate the relationship between the expression of the blue module lncRNAs and the OS of patients with HCC (Yang et al., 2018). LncRNAs with *p* < 0.05 was identified to have the prognostic value for HCC OS outcomes. Second, the lncRNAs with *p* < 0.001 in univariate Cox regulation analysis were further analyzed *via* the least absolute shrinkage and selection operator (LASSO) Cox regression analysis to select the most useful prognostic lncRNAs, called OS-related AR-lncRNAs, by using the glmnet package in R (Engebretsen and Bohlin, 2019). The “10-fold cross-validation” approach was used to facilitate parameter selection (Mao et al., 2019). Third, HCC patients were randomly divided into training set (186 cases) and testing set (184 cases). The data of the training set were used to generate the prognostic signature through forward conditional stepwise regression with multivariable Cox analysis using the OS-related AR-lncRNAs (Yang et al., 2018). A prognostic multi-lncRNA signature was conducted in which the risk score was calculated as follows: $risk\;score=\overset{n}{\underset{i=1}{\sum }}Coef_{i}\times x_{i}$ (*Coef*_*i*_ was the estimated regression coefficient derived from multivariate Cox regression analysis using the R/survival package (Huang et al., 2020), and *x*_*i*_ was the expression value of each selected AR-lncRNA). The median risk score in the training set was used as the cutoff point that divided HCC patients into a high-risk group and a low-risk group. Then, the formula was used to calculate the risk score of each HCC patient in the testing and whole set, followed by grouping them into high- and low-risk groups. Log-rank testing method was used to compare the differences of OS outcomes between the high- and low-risk groups *via* Kaplan–Meier survival analysis (Yang et al., 2018). The receiver operating characteristic (ROC) curve analysis in the “survivalROC” package (Heagerty and Zheng, 2005; Huang et al., 2017, 2020) was applied to examine the accuracy of the identified AR-lncRNA signature. Area under the curve (AUC) of 3-year OS outcomes based on the time-dependent ROC curves was used to compare the prediction accuracy of our newly identified AR-lncRNA signature with other three recently published signatures. The concordance index (C-index) was calculated to compare the prediction accuracy of prognostic signatures (Wang et al., 2017; Huo et al., 2020; Yang et al., 2020) by “survcomp” package (Schroder et al., 2011).

### Stratification Analysis

The whole set of patients was stratified by different infection type [HBV (*n* = 104) or HCV (*N* = 56)], alcoholic hepatitis (*n* = 117), age [≥60 years (*N* = 201) or <60 years (*n* = 169)], TNM stages [stage I and II (*n* = 256) or stage III and IV (*n* = 90)], α-fetoprotein (AFP) level [high: >300 (*n* = 65) or low: ≤300 (*n* = 212)]. The formula of risk score acquired in the training set was used to calculate the risk score of each HCC patient in each stratification cohort, followed by grouping them into high- and low-risk groups. Log-rank testing method was used to compare the differences of OS outcomes between the high- and low-risk groups via Kaplan–Meier survival analysis.

### Construction and Assessment of a Prognostic Nomogram

Multivariable Cox analysis was used to testify the prognostic independence of the AR-lncRNA signature where *p* < 0.05 was regarded as statistically significant. A forest plot was used to display the results of the multivariable Cox analysis. The R package rms (Chen S. et al., 2020) was used to construct the nomogram to assess the 3- and 5-year survival possibility for HCC patients. C-index was calculated to identify the discrimination of the nomogram (Huang et al., 2019). Calibration curve of the nomogram was generated to evaluate the consistency between its predicted values and the actual observed values by “nomogramEx” package (Du et al., 2020).

### Immunotherapy and Drug Responsiveness

The Tumor Immune Dysfunction and Exclusion (TIDE) tool (http://tide.dfci.harvard.edu/) was used to compute TIDE score for each tumor sample, which serves as a surrogate to predict the immunotherapy responsiveness (Jiang et al., 2018). The R package pRRophetic (Geeleher et al., 2014) (version 0.5) was applied for drug sensitivity prediction by using ridge regression to estimate the half-maximal inhibitory concentration (IC_50_) for each sample. Then, the prediction accuracy of drug sensitivity was evaluated by 10-fold cross-validation based on the Genomics of Drug Sensitivity in Cancer (https://www.cancerrxgene.org/) (Lui et al., 2020).

### Somatic Variants Analysis

Gene somatic mutation data with a total of 364 HCC samples based on the whole-exome sequencing platform of the TCGA-LIHC datasets were downloaded by TCGAbiolinks (Colaprico et al., 2016). Somatic variants analysis was performed by the R package maftools (Mayakonda et al., 2018) based on the TCGA-LIHC Mutect2 pipeline, which visualized the mutational signatures of the HCC cancer genome. Samples with frameshift insertions, missense mutations, multiple hits, non-sense mutations, splice-site mutations, frame shift deletions, in-frame insertions, or in-frame deletions were considered as positive for a mutation.

### Gene Set Enrichment Analysis

Genome-wide expression profiles of the HCC patients were subjected to gene set enrichment analysis (GSEA) (http://www.broad.mit.edu/gsea/) to analyze genes that were differentially expressed between the patients of the high- and low-risk groups (Yang et al., 2018). Gene sets used in this work were c2.cp.kegg.v7.0.symbols.gmt, which contained mainly Kyoto Encyclopedia of Genes and Genomes pathway and downloaded from the Molecular Signatures Database (MSigDB, http://software.broadinstitute.org/gsea/msigdb/index.jsp). Difference for which the NOM *p* < 0.05 was considered statistically significant.

### Immune Cell Infiltration

The immune cell infiltration status was acquired based on quanTIseq, a method to quantify the fractions of 10 immune cell types from bulk RNA-sequencing data (Finotello et al., 2019), by using the single-sample gene set enrichment approach (Zuo et al., 2020) to the transcriptomes of HCC.

### Correlation Between the Expression Levels of Selected lncRNAs and ARGs

All of the expression data of ARGs of HCC patients were normalized by log2 transformation. Pearson correlation analysis was applied to calculate the correlation between the signature-involved lncRNAs and ARGs. An ARG with a |correlation coefficient| >0.3 and *p* < 0.05 was considered to be the putative target of a lncRNA. The lncRNAs-ARG coexpression network was presented by Cytoscape (version 3.6.2). Correlation scatter plots of each paired lncRNA and ARG were shown for the whole set of HCC patients.

### Statistical Analysis

All dataset analyses were performed using R software (version 3.5.1). The association between each clinical trait and each module was determined using the χ^2^ test, Wilcoxon rank sum test, or unpaired *t*-test according the data type of each clinical trait. Univariate and multivariate Cox regression was used to assess prognostic significance. Kaplan–Meier and log-rank tests were used to perform survival analysis. The Student *t*-test was used to compare two independent groups. Mean ± standard deviation with statistical significance was set at *p* < 0.05.

## Results

### Identification of AR-lncRNA Modules for HCC by WGCNA

**Figure 2:**
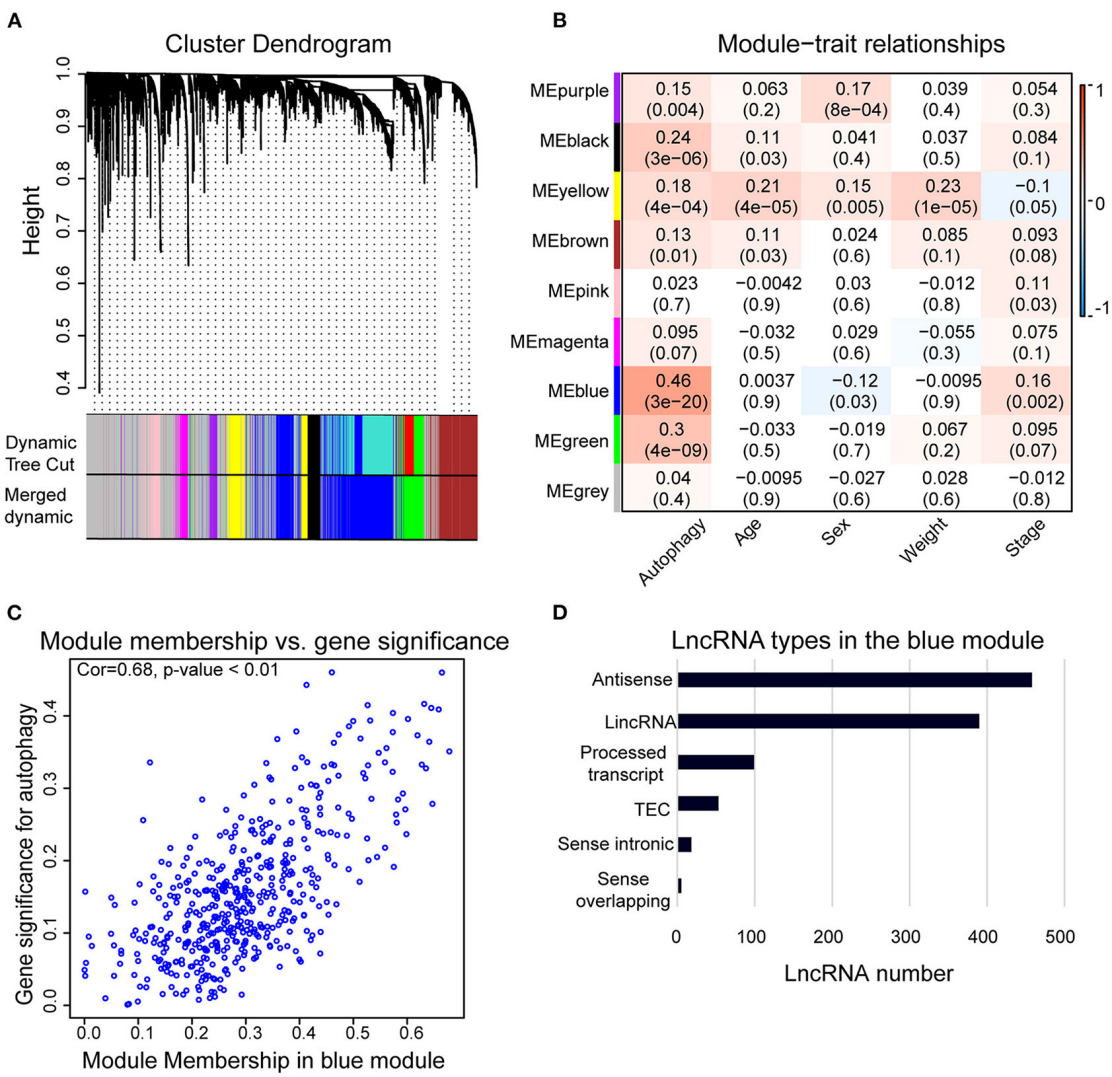
Weighted correlation network analysis (WGCNA) of lncRNAs in hepatocellular carcinoma (HCC) samples. **(A)** Gene clustering tree (dendrogram) obtained by hierarchical clustering of adjacency-based dissimilarity. **(B)** Module–trait relationships. Each row corresponds to a module of eigengenes, and each column corresponds to a clinical feature, respectively. Each cell contains the corresponding value of correlation in the first line and *p*-value in the second line, respectively. The cell color presents the correlation according to the color legend. **(C)** Correlation between membership in the blue module and membership in the autophagy pathway of the eigengenes in blue module by Pearson correlation analysis. Cor, correlation coefficient. **(D)** Bar chart shows the types of lncRNAs in the blue module.

We used WGCNA to analyze the lncRNAs detected in HCC samples. The dynamic tree cutting method was used to cluster the lncRNAs in layers, and then highly similar modules were merged (Figure 2A). Coexpression network by WGCNA analysis revealed that the 3,889 lncRNAs in HCC sample were grouped into nine modules (Supplementary Table 2). The highest association between lncRNA modules and clinical traits was found between the blue module and autophagy (*r*^2^ = 0.46, *p* < 0.05). The blue module was negatively correlated to the sex of HCC patients (*r*^2^ = −0.12, *p* < 0.05) and positively correlated to the stage of HCC patients (*r*^2^ = 0.16, *p* < 0.05), respectively (Figure 2B). The correlation coefficient between memberships in blue module and memberships in the autophagy pathway of eigengenes in the blue module was 0.68 (*p* < 0.001) (Figure 2C), indicating indeed a relationship between the blue module and autophagy activity. Therefore, we defined the blue module as AR-lncRNA module. There were 1,023 lncRNAs in the AR-lncRNA module, among which 44.8% was antisense lncRNAs, and 38.1% was long intergenic non-coding RNAs (Figure 2D).

### Establishment and Verification of AR-lncRNA Prognostic Signature for HCC

As previously reported, autophagy pathway is associated with the prognosis of HCC patients (Zhu et al., 2020b); univariate Cox regression analysis was used to explore the relationship between AR-lncRNAs and HCC prognosis. The data revealed that 354 among the total of 1,023 AR-lncRNAs (34.6%) performed the capacity of prognosis for HCC OS outcomes (*p* < 0.05) (Supplementary Table 1), indicating a critical role of AR-lncRNAs in HCC prognosis. Then, the LASSO Cox regression analysis identified seven OS-related AR-lncRNAs from the above identified 354 lncRNAs with univariate Cox regression, *p* < 0.001 (Supplementary Figure 2). Conformably, high expression of these seven OS-related AR-lncRNAs predicted a poor prognosis of HCC patients.

**Figure 3:**
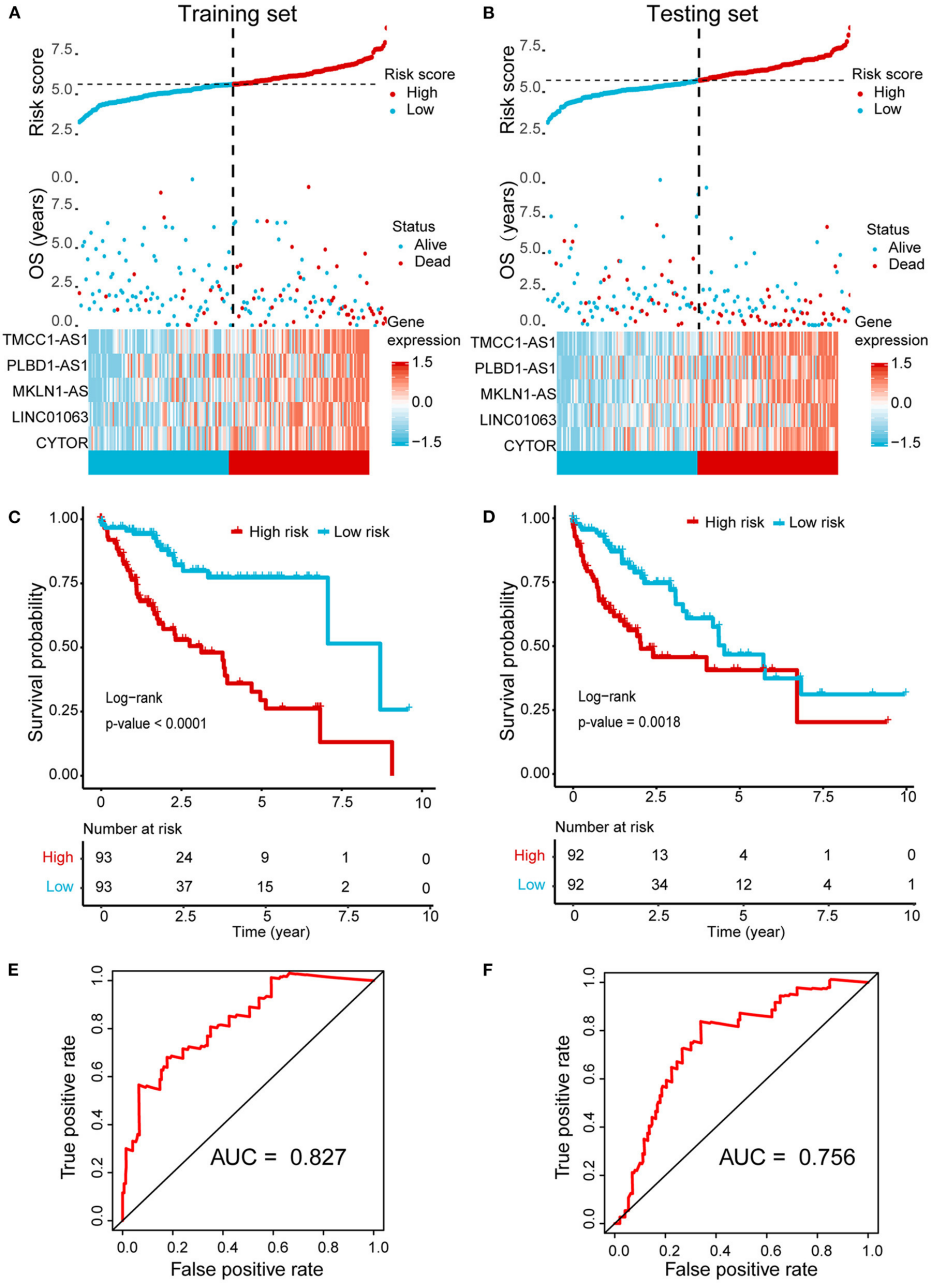
Establishment and validation of a five-AR-lncRNA prognostic signature for HCC survival prediction. Patients with a high or a low expression of the selected five AR lncRNAs have significantly different survival probability. **(A,B)** The distribution of risk score, survival state, and expression heatmap of the selected five AR lncRNAs in the training set **(A)** and testing set **(B)**, respectively. **(C,D)** Kaplan–Meier survival curve for the high- and low-risk groups divided by the cutoff value in the training set **(C)** and testing set **(D)**, respectively. *p*-values were obtained via log-rank test. **(E,F)** The receiver operating characteristic curve (ROC) for the prognosis prediction of the signature at 3 years of overall survival (OS) in the training set **(E)** and testing set **(F)**, respectively.

Next, we randomly divided the HCC patients in TCGA data into training set (186 cases) and testing set (184 cases). The above identified seven OS-related AR-lncRNAs were used for the prognostic module building by the forward conditional stepwise regression with multivariable Cox analysis in the training set. An AR-lncRNA signature, composed of five feature lncRNAs (TMCC1-AS1, PLBD1-AS1, MKLN1-AS, LINC01063, and CYTOR), was constructed for HCC prognosis. The risk score of each sample in the training set was calculated according to the expression of the five AR lncRNAs by using the following formula: risk score = CYTOR expression × 0.17456 + LINC01063 expression × 0.30093 + MKLN1-AS expression × 0.27462 + PLBS1-AS1 expression × 0.17218 + TMMC1-AS1 expression × 0.28974, in which the coefficients were derived from forward conditional stepwise regression with multivariable Cox analysis. Then, the risk scores were ranked from low to high. According to the cutoff point using the median risk score (cutoff = 0.958887), patients in training set were divided into a high-risk group (93 cases, risk score ≥0.958887) and a low-risk group (93 cases, risk score <0.958887). The high-risk group showed higher expression of these five AR lncRNAs and had a poor living status and significantly shorter OS (log-rank test, *p* < 0.0001), compared with the low-risk group (Figures 3A,C). The median survival time for high- and low-risk patients was 2.5 and 7 years, respectively. The same formula was applied to the testing set and revealed a similar finding as that in the training set (Figures 3B,D). Of note, the AUC values of ROC curve of 3-year OS were 0.827 and 0.756 in the training set and testing set, respectively, indicating good sensitivity and specificity of this five-AR-lncRNA signature in predicting the survival rate of HCC patients (Figures 3E,F).

**Figure 4:**
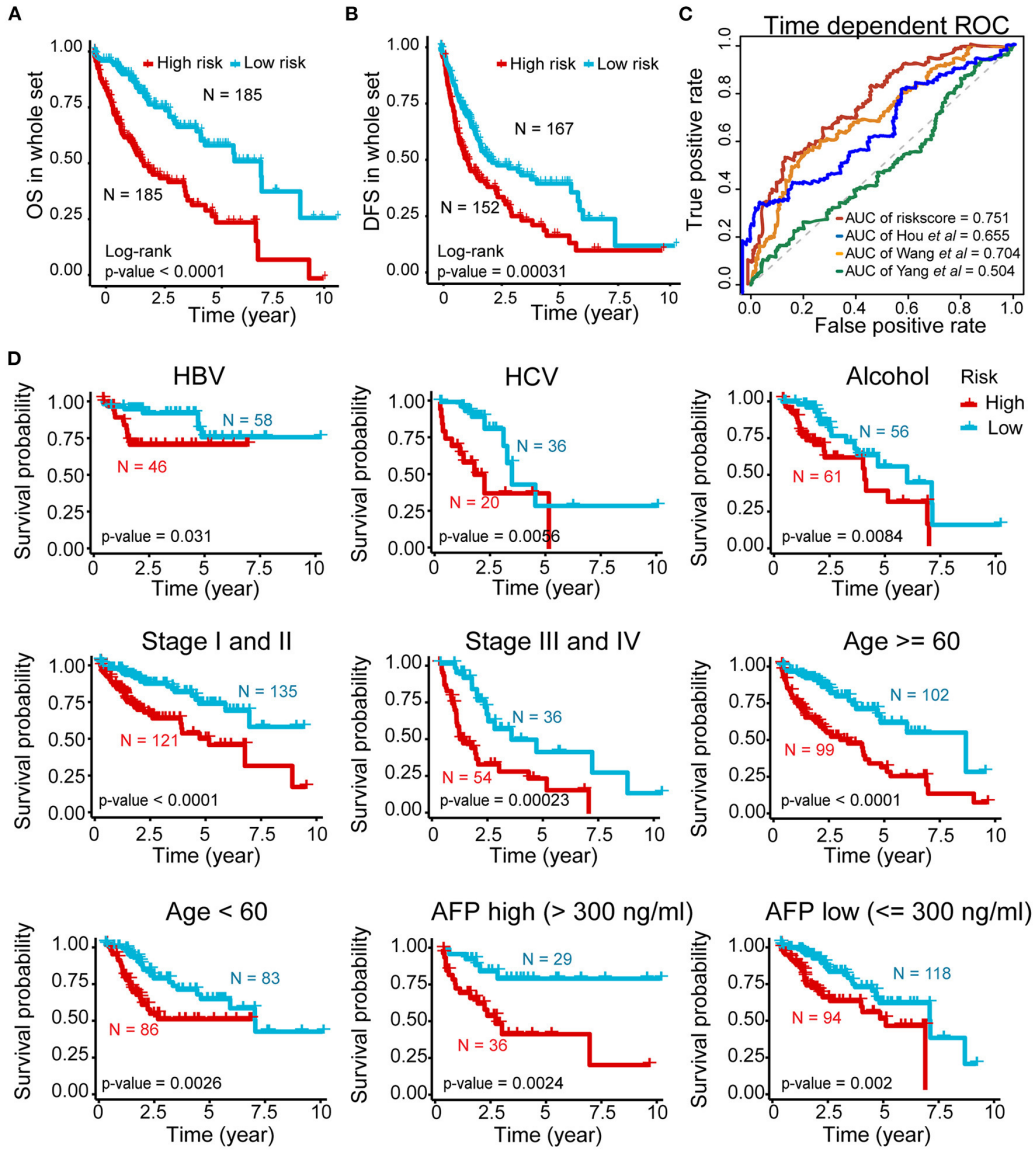
Prognostic power of the five-AR-lncRNA prognostic signature in stratification analysis. **(A)** Kaplan–Meier survival curve for the high- and low-risk groups divided by the cutoff value in the whole set. **(B)** Disease-free survival (DFS) for the high- and low-risk patients. **(C)** The ROC analysis of OS outcomes for the five-AR-lncRNA signature (TMCC1-AS1, PLBD1-AS1, MKLN1-AS, LINC01063, CYTOR) by us; five-autophagy-related gene (ARG) signature (HDAC1, RHEB, ATIC, SPNS1, and SQSTM1) by Huo et al. (2020); four-lncRNA signature (ENSG00000234608, ENSG00000242086, ENSG00000273032, ENSG00000228463) by Yang et al. (2020) and four-lncRNA signature (RP11-322E11.5, RP11-150O12.3, AC093609.1, CTC-297N7.9) by Wang et al. (2017) **(D)** Kaplan–Meier curves of patients stratified by different clinicopathological traits. Hepatitis B virus, HBV; hepatitis C virus, HCV. *p*-values were obtained via log-rank test.

Further validation showed ideal distinction of 3-year OS outcomes between the high- and low-risk groups of the whole set (*p* < 0.0001) (Figure 4A). The prognostic power of our signature was also confirmed by good prediction effectiveness on the DFS of all HCC patients (*p* < 0.001) (Figure 4B).

Three previously published HCC-related signatures derived from TCGA successfully and significantly predict the OS outcomes, including five-ARG signature (HDAC1, RHEB, ATIC, SPNS1, and SQSTM1) by Huo et al. (2020) four-lncRNA signature (ENSG00000234608, ENSG00000242086, ENSG00000273032, ENSG00000228463) by Yang et al. (2020) and four-lncRNA signature (RP11-322E11.5, RP11-150O12.3, AC093609.1, CTC-297N7.9) by Wang et al. (2017). To compare the sensitivity and specificity of our five-AR-lncRNA signature for the prognosis prediction with these three existing signatures, we performed the time-dependent ROC analysis (Liao et al., 2020). The AUCs of 3-year OS for Hou's signature, Yang's signature, and Wang' signature was 0.655, 0.504, and 0.704, respectively, all of which were lower than that of our five-AR-lncRNA signature (AUC = 0.751) (Figure 4C). This indicated an obvious improvement in the estimation of survival rate of HCC patients achieved by this five-AR-lncRNA signature, which can also be seen from the restricted mean survival curve [C-index: 0.71 vs. 0.66 (*p* < 0.05), 0.51 (*p* < 0.001), and vs. 0.67 (*p* < 0.05)] (Supplementary Figure 3).

### Stratification Analysis Based on the AR-lncRNA Prognostic Signature

In order to explore the applicability of the five-AR-lncRNA signature, we next performed the stratification analysis. Patients from the whole set were stratified by different infection type (HBV, HCV, alcoholic hepatitis), age (≥60 or <60 years), TNM stages (stage I and II or stage III and IV), and AFP level (>300 or ≤ 300). Each subgroup was then divided into a high- and low-risk groups based on the median risk score derived from the training set. Kaplan–Meier curves showed that, for all subgroups, the high-risk group had a significant poorer survival rate than that in the low-risk group (*p* < 0.05) (Figure 4D). This indicated that the five-AR-lncRNA signature could accurately predicate the prognosis of HCC patients regardless of different clinical traits.

### Establishment of a Nomogram for HCC Prognosis Based on Independent Prognostic Factors

**Figure 5:**
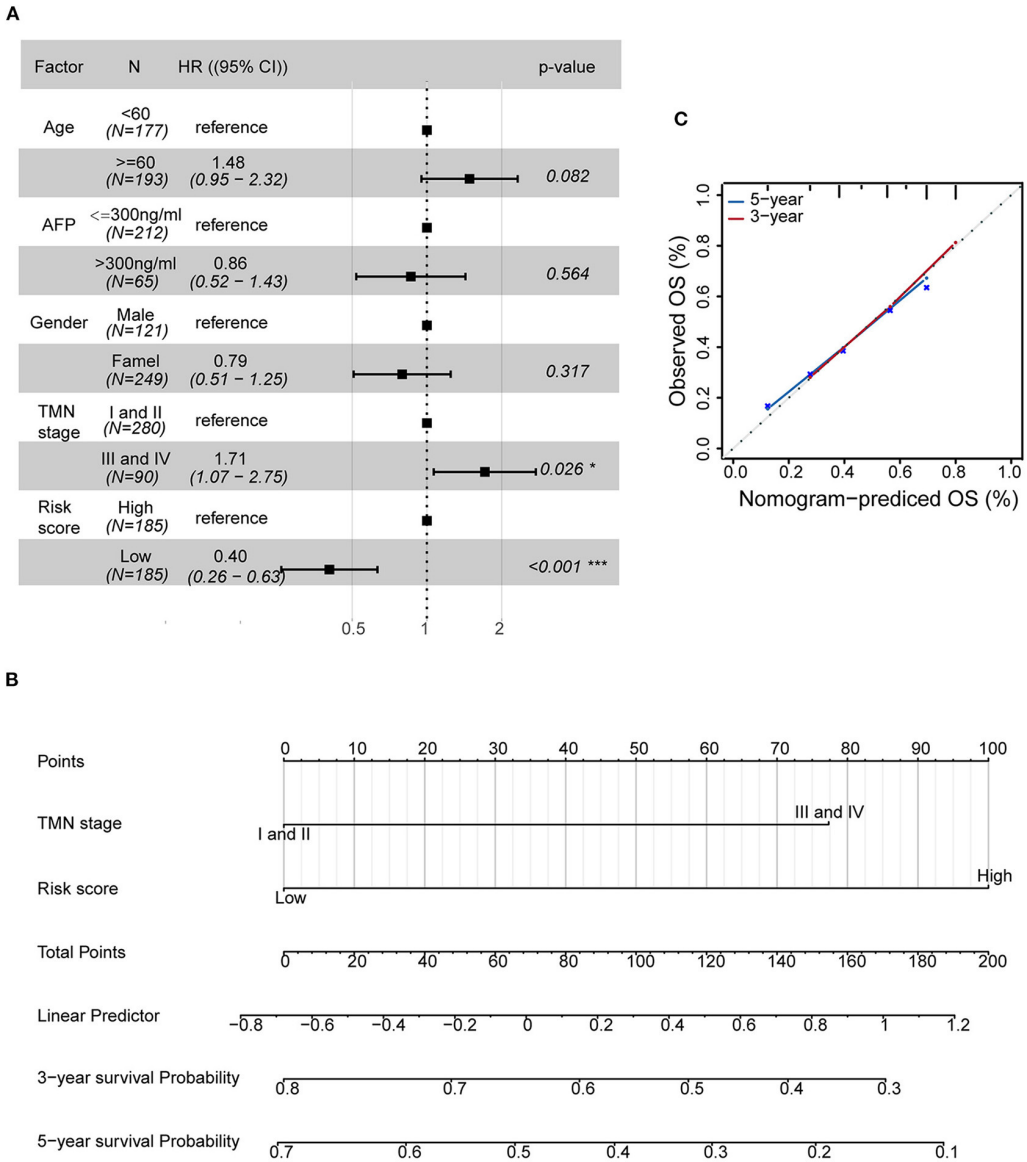
Establishment of a nomogram containing independent factors of OS prediction. **(A)** The forest plot based on clinical characteristics and the five-AR-lncRNA signature by multivariate Cox regression analysis. CI, confidence interval; HR, hazard ratio. **(B)** A prognostic nomogram predicting 3- and 5-year OS outcomes of HCC based on TNM stage and the risk score of the five-AR-lncRNA signature. **(C)** Calibration curves for the nomogram of 3- (red) and 5-year (blue) OS prediction for the whole set of HCC patients.

To examine the importance of the five-AR-lncRNA signature when considering other conventional clinical characteristics, we carried out the multivariate Cox regression analysis. The results revealed that after adjusting for other factors, the risk score of the five-AR-lncRNA signature served as an independent factor for the prognosis of HCC (*p* < 0.001) (Figure 5A). Among the other clinical characteristics, only TNM stage can act as an independent prognostic factor (*p* < 0.05) (Figure 5A).

The prognostic nomogram can assist individualized survival prediction and guide treatment strategies (Wang et al., 2019). Therefore, we used the above selected independent prognostic factors, including TNM stage and the risk score of the five-AR-lncRNA signature, to construct a prognostic nomogram for the 3- and 5-year OS prediction of each individual HCC patient (Figure 5B). The calibration curves showed an agreement between the predicted survival and actual survival (Figure 5C), and the C-index of nomogram reached 0.745 (95% confidence interval, 0.686–0.805), highlighting an ideal predictive value of our nomogram. So far, we facilitated the utilization of the five-AR-lncRNA signature for HCC.

### Therapeutic-Outcomes Analysis for the High- and Low-Risk Groups

**Figure 6:**
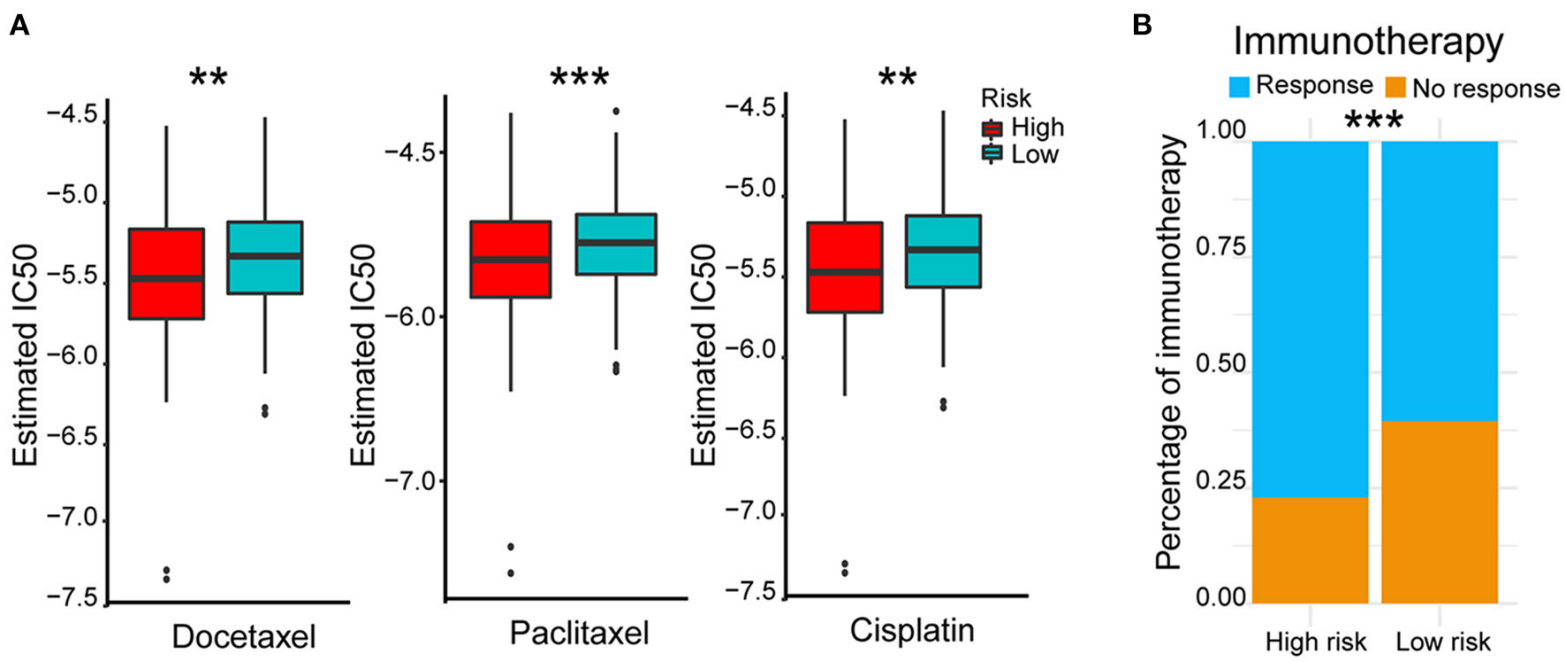
Therapeutic outcomes of the high- and low-risk groups based on the five-AR-lncRNA signature. **(A)** Sensitivity to different chemotherapeutic drugs, docetaxel (left), paclitaxel (middle) and cisplatin (right), in the high- and low-risk groups. *p*-values were obtained via Wilcoxon rank sum test. **(B)** Response to immunotherapy in the high- and low-risk groups. *p*-values were obtained via Fisher exact test. **p < 0.01 and ***p < 0.001, respectively.

In view of the survival differences between the high- and low-risk groups, we speculated that their responses to different treatments were different. Thus, we further analyzed different therapeutic outcomes of HCC patients, including chemotherapeutic responsiveness and immunotherapy sensitivity. HCC patients in the low-risk group showed stronger drug sensitivity to chemotherapy according to their lower 50% inhibiting concentration (IC_50_) of docetaxel, paclitaxel, and cisplatin (*p* < 0.01) (Figure 6A) and better responses to immunotherapy (*p* < 0.001) (Figure 6B). These results indicated that the five-AR-lncRNA signature also had a certain degree of separability on the therapeutic response of HCC patients.

### GSEA, Somatic Variants Analysis, and Immune Infiltration for the High- and Low-Risk Groups

**Figure 7:**
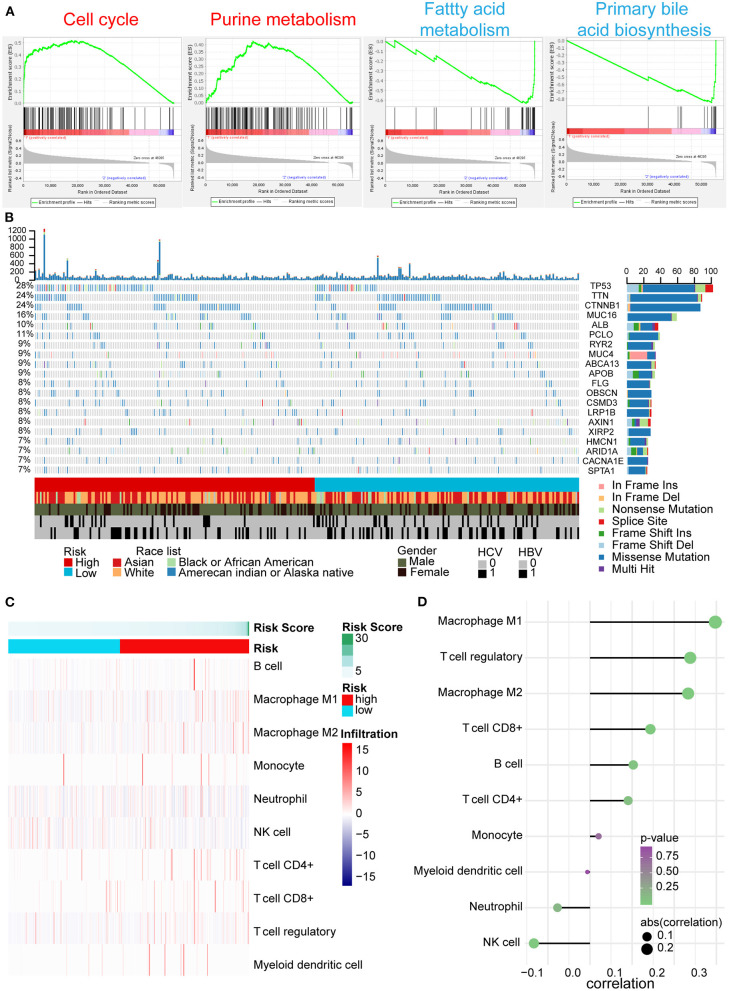
GSEA, gene mutation analysis, and immune infiltration of the high- and low-risk groups based on the five-AR-lncRNA signature. **(A)** GSEA of the differentially expressed genes between the high-risk group (marked in red) vs. low-risk group (marked in blue). For each group, only two most significantly enriched functional gene sets are shown. **(B)** The genomic landscape and mutational signatures of 307 HCC patients (84.34% of 364 samples). Individual tumor mutation rates are shown in the top panel, whereas the risk, race, gender, and HCV and HBV infection status of HCC patients are detailed in turn in the bottom panel. The middle panel shows genes with statistically significant levels of mutation (MutSig suite, FDR <0.1). The name and type composition of each mutant gene are shown on the right, and mutation types are indicated in the legend at the bottom, respectively. **(C)** Association between risk score and immune cell population in low- and high- risk groups. **(D)** The bubble map shows the correlation between risk score and immune cell subset infiltration. *X* and *Y* axes represent the correlation coefficient and the type of infiltrated immune cell subset, respectively. The color of each bubble shows the *p*-value of correlation, whereas the size shows the absolute value of correlation coefficient.

According to the above results, we further explored the potential explanations for this five-AR-lncRNA signature, which can distinguish the differences of survival and therapeutic outcome from HCC patients. GSEA results revealed that the high-risk group showed gene enrichment in cell cycle and purine metabolism pathways (*p* < 0.05) (Supplementary Table 3). The role of the former was well-established in cancer proliferation, invasion, and metastasis (Otto and Sicinski, 2017), whereas the latter was confirmed one of the markers for liver cancer as it promoted the progression of liver cancer (Chong et al., 2020). In contrast, the low-risk group showed gene enrichment in primary bile acid biosynthesis and fatty acid metabolism pathways (*p* < 0.05) (Supplementary Table 4), both of which were usually down-regulated in HCC patients (Wang et al., 2016) (Figure 7A).

Somatic variants analysis showed the top 20 mutated genes of HCC samples, including TP53, CTNNB1, ALB, AXIN1, and ARID1A (Figure 7B), which was concordant with the previously reported results (Cancer Genome Atlas Research Network. Electronic address and Cancer Genome Atlas Research, 2017). We found a significantly higher frequency of TP53 mutation in the high-risk group than that in the low-risk group (Figure 7B).

In tumor microenvironment, what constitutes immune cell subsets affects the antitumor effects of immunotherapy (Bao et al., 2019). With the increase in risk score, the ratio of M1, M2 macrophages, and regulatory T (Treg) cells increased markedly (*p* < 0.05); especially the ratio of M1 cells infiltration showed positive correlation with risk score (correlation coefficient = 0.3), whereas the ratio of natural killer (NK) cells decreased significantly accompanied by the increase of risk score (*p* < 0.05) (Figures 7C,D).

### Construction of the Five AR lncRNAs and ARGs Coexpression Network

**Figure 8:**
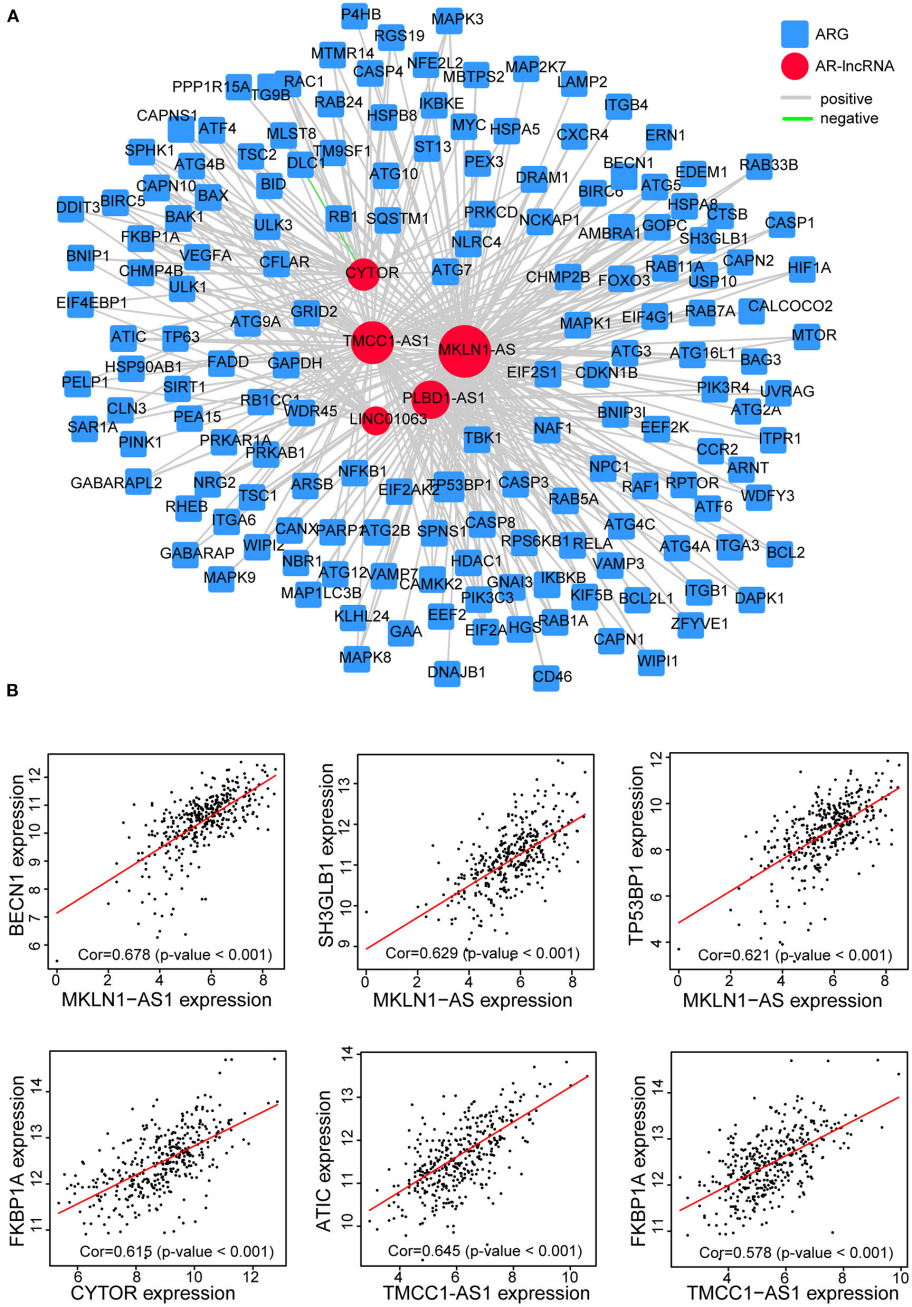
Establishment of the five-AR-lncRNA-ARG coexpression network in HCC. **(A)** LncRNAs-ARG coexpression network was established among five signature-involved lncRNAs and ARGs. Each red round node stands for one AR-lncRNA, whereas each blue square node stands for one ARG. Within this coexpression network, gray line represents positive association pair, whereas green line represents negative association pair. The size of each round node is proportional to the number of ARG genes it correlated with. The thickness of a line represents the correlation coefficient. **(B)** Representative correlation scatter plots of ARGs and the five AR lncRNAs by Pearson correlation analysis.

Moreover, by constructing the lncRNAs-ARG coexpression network, we found an arsenal of ARGs correlated with the five AR lncRNAs (*p* < 0.05, |correlation coefficient| >0.3) (Supplementary Table 5) and the largest number of ARGs coexpressed with MKLN1-AS1 (Figure 8A). For instance, BECN1, a central protein triggering the autophagy protein cascade (Han et al., 2018), coexpressed with more than two selected AR-lncRNAs and displayed the highest correlation coefficient with MKLN1-AS1 (correlation coefficient = 0.678). The other reported ARGs, including FKBP1A, TP53BP1, and SH3GLB1 (Ge et al., 2014; Wild et al., 2016; Sharma et al., 2018), also showed significant expression correlation with some of these AR-lncRNAs (correlation coefficient >0.6, *p* < 0.001) (Figure 8B). The above evidence elucidated that these five AR lncRNAs in our signature may affect the prognosis of HCC patients *via* regulating ARGs expression.

## Discussion

Prognostic models for HCC based on lncRNAs have been reported continuously (Wang et al., 2017; Yang et al., 2020). However, because of the heterogeneity of each cohort and different analysis methods of each study, the predictive effectiveness of their results is not universally applicable (Liu et al., 2016). In this current study, we for the first time achieved a five-AR-lncRNA signature of HCC prognosis. This five-AR-lncRNA signature can be applied to all TCGA-HCC patients even classified by a variety of sorting schemes (including different etiology, TNM stage, age, and AFP level). It showed ideal distinctions of OS outcomes between the high- and low-risk groups with good AUC values. In addition, the accuracy of our five-AR-lncRNA signature also surpassed that of three recently reported prediction signature of HCC based on ARGs or OS-related lncRNAs (Wang et al., 2017; Huo et al., 2020; Yang et al., 2020). The nomogram model, consisting of the TNM stages and the risk score derived from the five-AR-lncRNA signature, can visually predict the 3- and 5-year OS outcomes for individual HCC patient. Of note, the five-AR-lncRNA signature was also able to identify significant differences in chemotherapeutic and immunotherapy responses for HCC patients.

Our five AR lncRNAs include TMCC1-AS1, PLBD1-AS1, MKLN1-AS, LINC01063, and CYTOR, all of which were highly expressed in the high-risk group with poor OS. Consistent with our findings, previous studies have shown that HCC patients with higher expression of TMCC1-AS1 showed shorter OS when compared with the low-expression ones (Zhao et al., 2018; Deng et al., 2020). CYTOR has been reported as an adverse factor of pan cancers *via* promoting proliferation, migration, invasion, metastasis, and drug resistance of tumor cells (Wang et al., 2018; Zhang and Li, 2018; Liu et al., 2019a; Zou et al., 2019; Chen W. et al., 2020; Zhu et al., 2020a). MKLN1-AS has also been reported to be a risk factor of liver cancer (Xiao et al., 2019), although it is also reported as a protective lncRNA of HCC in HBV-positive patients, which might due to the analysis being restricted to HBV-HCC (Zhao et al., 2020). Despite this, our signature could also effectively distinguish the different OS outcomes of high- and low-risk groups of HBV-HCC patients. In addition, we also contributed two new risk lncRNAs, LINC01063 and PLBD1-AS1, for HCC. Our data showed that the expression levels of PLBD1-AS1 and LINC01063 were correlated with tumor suppressor p53-binding protein 1 (TP53BP1) (correlation coefficient = 0.44), and charged multivesicular body protein 4B (CHMP4B) (correlation coefficient = 0.36), respectively. TP53BP1 recruitment can mediate the activation of autophagy by tumor self-DNA damage response (Sharma et al., 2018). CHMP4B recruitment is important at a late step of mitophagosome formation (Zhen et al., 2020). Further studies are warranted to confirm the definite role and mechanism of LINC01063 and PLBD1-AS1 in the development of HCC regarding an autophagy mechanism.

Preliminary mechanism explorations by GSEA and somatic variants analysis revealed that dysregulated cell cycle, purine metabolism, and TP53 mutation may play important roles resulting in poor OS outcomes of HCC patients in the high-risk group. Consistent with these findings, CYTOR has been reported to promote cyclin D1 expression, which regulated G1-to-S phase progression and formed active complexes that promoted cell cycle progression (Alao, 2007), resulting in inhibition of cell apoptosis (Galamb et al., 2020). TMCC1-AS1 correlated with AICAR transformylase (ATIC) (correlation coefficient = 0.61), an autophagy-related protease that catalyzed the last two steps in the purine biosynthesis pathway (Huo et al., 2020). The mutation of TP53, which was proven the most common mutation in patients with liver cancer, showed higher frequency in the high-risk group. Previous study has reported that TP53 inhibited autophagy by inhibiting AMPK but activating mTOR signaling pathway (Zhao et al., 2020). As such, it is interesting to further explore the casual role between TP53 mutation and dysregulated autophagy during HCC development.

Prediction of therapeutic response is helpful for the precision medicine in cancer treatment, especially for the selection of the first line of treatments that determines the prognosis of cancer patient. Our data showed that the five-AR-lncRNA signature was also able to identify significant differences between chemotherapeutic and immunotherapy responses. The low-risk group was more sensitive to cisplatin, docetaxel, and paclitaxel, which were frequently used chemotherapeutic drugs for HCC (Jin et al., 2018; Sung et al., 2020; Tekchandani et al., 2020). This might because of the different autophagy activities between the high- and low-risk groups. Previous studies have demonstrated that high levels of autophagy activity led to reduced sensitivity of hepatocellular carcinoma cells to chemotherapeutic drugs (Xiong et al., 2017). Moreover, the infiltration and function of immune cells in the tumor microenvironment could be weakened by the activation of autophagy-related signaling pathways in tumor microenvironment, which may also affect the efficiency of immunotherapy; for example, tumor cell autophagy weakens the killing function of NK cells (Huang et al., 2018; Yao et al., 2018). M2 cells and Treg cells, serving as immunosuppressive cells, played negatively prognostic role in HCC (Fridman et al., 2017). Consistently, in this current study, with the increase of risk score, NK cell infiltration decreased significantly, whereas the ratio of M2 macrophages and Treg cells significantly increased. Although the ratio of M1 macrophages, a positive factor for the prognosis of HCC (Fridman et al., 2017), increased with the increase of risk score, its role might be overwhelmed by above negative ones. In a word, the relationship among tumor autophagy and immune infiltration implied that regulating the infiltration of innate and acquired immune cells by controlling the level of autophagy may be a novel strategy to improve antitumor immunotherapy of HCC.

Collectively, our five-AR-lncRNA signature can predict not only the OS outcomes, but also the therapeutic response of HCC patients. Prognostic nomogram, using clinical TNM stages and the risk score of the five-AR-lncRNA, provides a firsthand prognostic tool for HCC patients. Therefore, further validations in other independent cohorts and mechanism studies will provide solid evidence to apply this five-AR-lncRNA signature as a clinical index of HCC precise treatment and prognosis.

## Data Availability Statement

The datasets presented in this study can be found in online repositories. The names of the repository/repositories and accession number(s) can be found in the article/Supplementary Material.

## Author Contributions

XD designed and performed the experiments, analyzed the data, and wrote the manuscript. QB, SC, XC, SL, ZZ, and WG performed the analysis. XL designed the research, supervised the study, and wrote the paper. YD and YY devised the concept, designed the research, and wrote the paper. All authors contributed to the article and approved the submitted version.

## Conflict of Interest

The authors declare that the research was conducted in the absence of any commercial or financial relationships that could be construed as a potential conflict of interest.

## Supplementary Material

Supplementary Figure 1 Determination of parameter β of the adjacency function in the WGCNA algorithm.

Supplementary Figure 2 Screening for AR-lncRNAs which related to OS outcomes of HCC. **(A)** Least absolute shrinkage and selection operator (LASSO) regression coefficient profiles of AR-lncRNAs in the blue module. Seven AR-lncRNAs were selected out. **(B–H)** Kaplan-Meier survival curve for patients with a high- or low-expression of the seven AR-lncRNAs which were closely associated with overall survival (OS) of HCC patients (*p*-value < 0.01).

Supplementary Figure 3 Comparison of the five-AR-lncRNA signature with three published prognostic signatures for HCC. Restricted mean survival (RMS) curves and concordance index (C-index) for the five-AR-lncRNA signature and other published signatures including five-ARG signature by Huo et al. (2020) **(A)**, 4-lncRNA signature by Yang et al. (2020) **(B)** and 4-lncRNA by Wang et al. (2017) **(C)**, respectively.

Supplementary Table 1 Univariate Cox regression analysis of the AR-lncRNAs significantly related to HCC OS outcomes.

Supplementary Table 2 Gene list of different modules of lncRNAs based on WGCNA in HCC.

Supplementary Table 3 Detailed enrichment results of GSEA for high-risk group based on the AR-lncRNAs prognostic signature.

Supplementary Table 4 Detailed enrichment results of GSEA for low-risk group based on the AR-lncRNAs prognostic signature.

Supplementary Table 5 The co-expression network established from five AR-lncRNAs and ARGs in HCC.
