# Supplementary figures and images for: Identification of a Five-Autophagy-Related-lncRNA Signature as a Novel Prognostic Biomarker for Hepatocellular Carcinoma
Source: Front Mol Biosci. 2021 Jan 11;7:611626. doi: 10.3389/fmolb.2020.611626 (PMC7831610; doi:10.3389/fmolb.2020.611626)

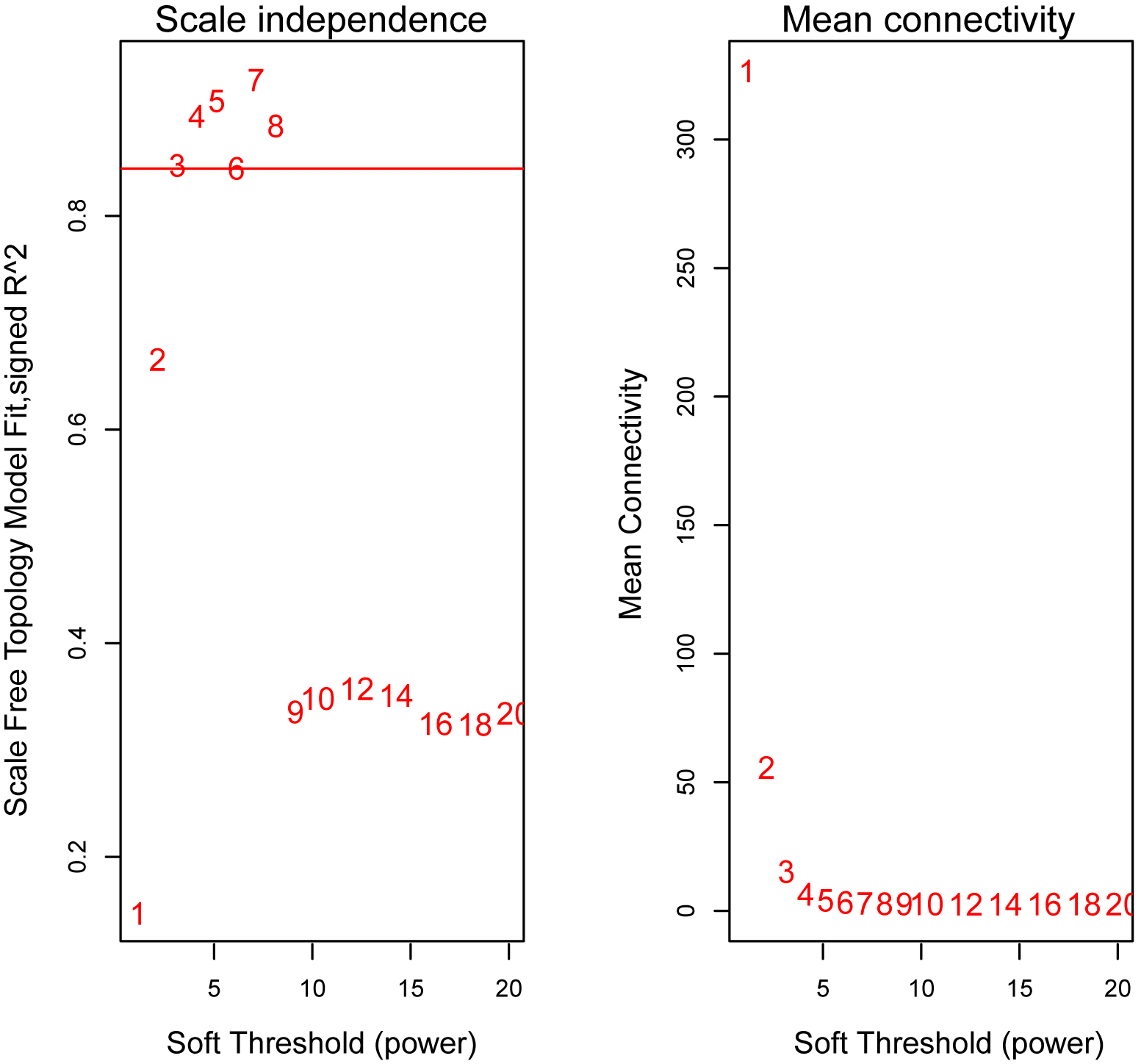

Supplement: Supplementary Figure 1 — Determination of parameter β of the adjacency function in the WGCNA algorithm. [file Image_1.TIF]

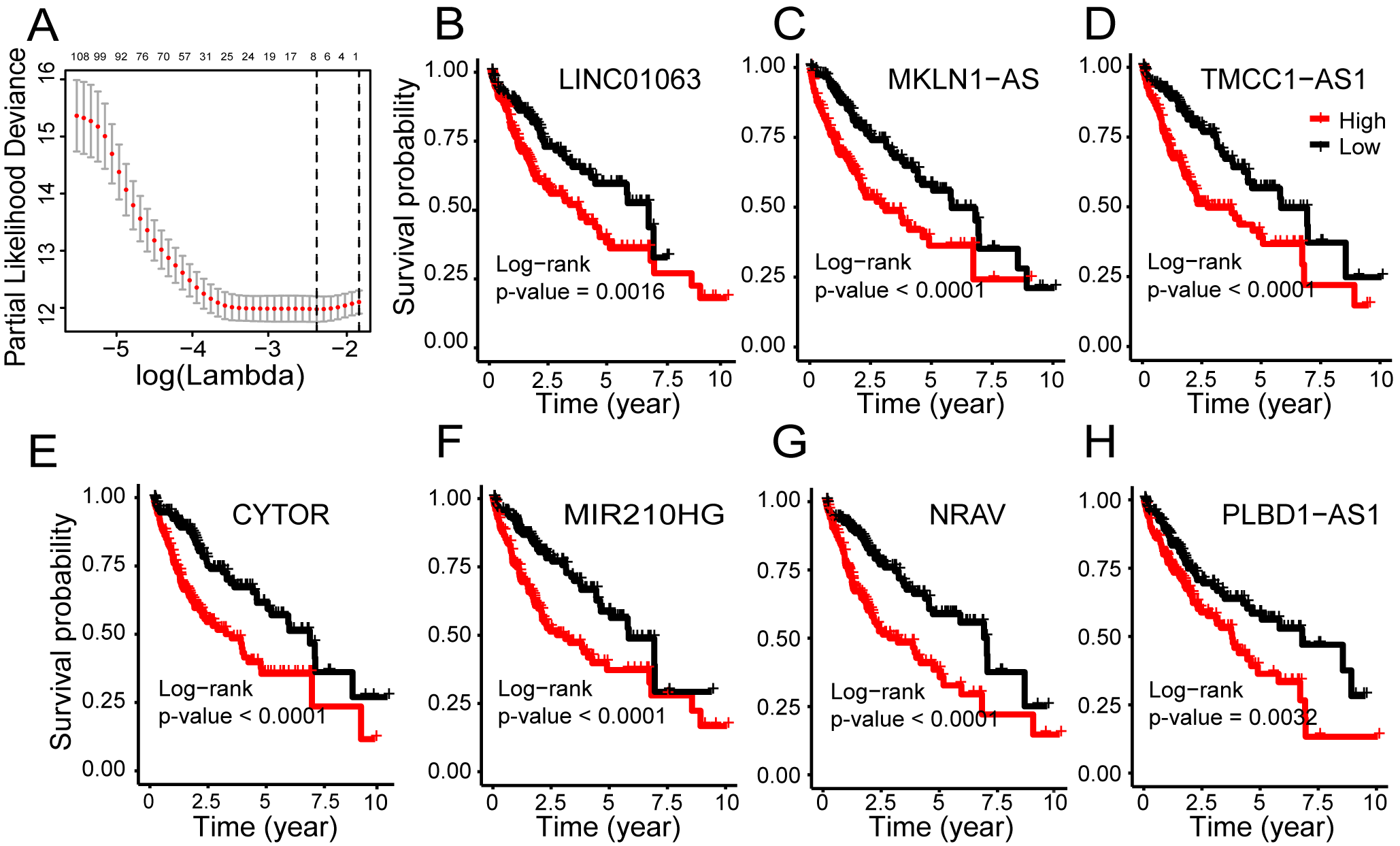

Supplement: Supplementary Figure 2 — Screening for AR-lncRNAs which related to OS outcomes of HCC. (A) Least absolute shrinkage and selection operator (LASSO) regression coefficient profiles of AR-lncRNAs in the blue module. Seven AR-lncRNAs were selected out. (B–H) Kaplan-Meier survival curve for patients with a high- or low-expression of the seven AR-lncRNAs which were closely associated with overall survival (OS) of HCC patients (p-value < 0.01). [file Image_2.TIF]

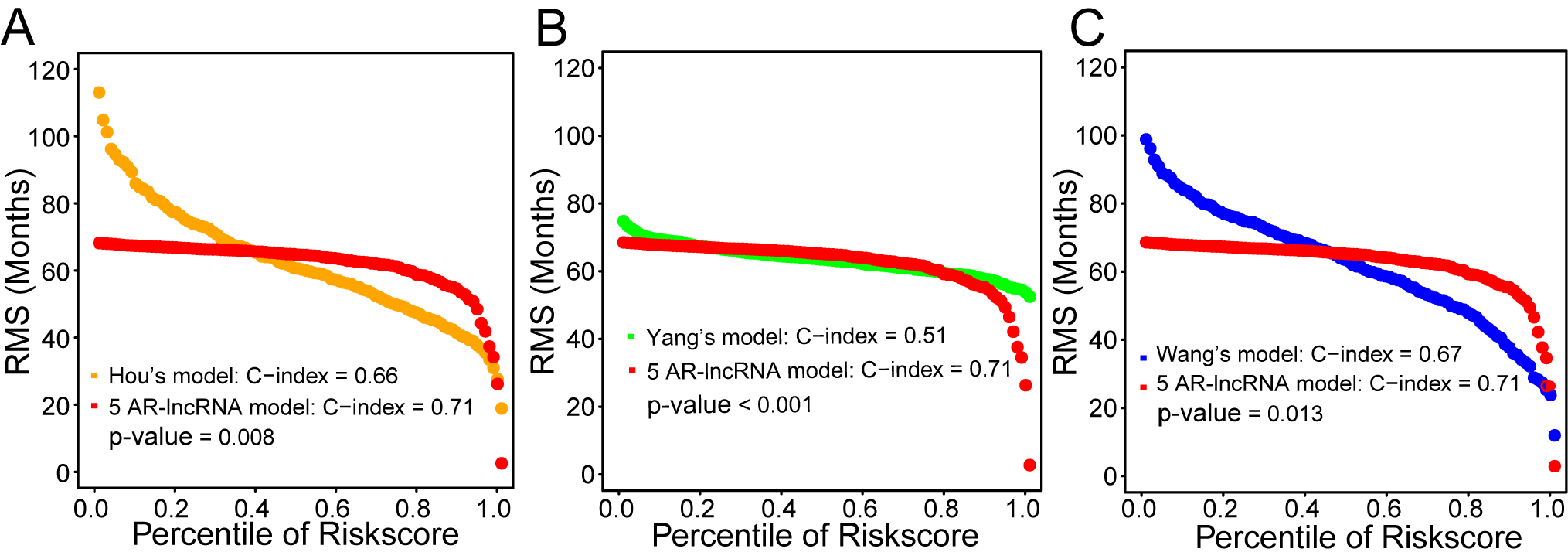

Supplement: Supplementary Figure 3 — Comparison of the five-AR-lncRNA signature with three published prognostic signatures for HCC. Restricted mean survival (RMS) curves and concordance index (C-index) for the five-AR-lncRNA signature and other published signatures including five-ARG signature by Huo et al. (2020) (A), 4-lncRNA signature by Yang et al. (2020) (B) and 4-lncRNA by Wang et al. (2017) (C), respectively. [file Image_3.TIF]
